# Supplementary material for: Hollow Mesoporous Silica Nanoparticles as a New Nanoscale Resistance Inducer for Fusarium Wilt Control: Size Effects and Mechanism of Action
Source: Int J Mol Sci. 2024 Apr 20;25(8):4514. doi: 10.3390/ijms25084514 (PMC11050273; doi:10.3390/ijms25084514)
Supplement: Supplementary file 1 [file ijms-25-04514-s001.zip › ijms-2913222-supplementary.pdf]

Supplementary Materials

Hollow Mesoporous Silica Nanoparticles as a New Nanoscale Resistance Inducer for Fusarium Wilt Control: Size Effects and Mechanism of Action

Chaopu Ding †, Yunfei Zhang †, Chongbin Chen, Junfang Wang, Mingda Qin, Yu Gu, Shujing Zhang *,
Lanying Wang and Yanping Luo *

School of Tropical Agriculture and Forestry, Hainan University, Haikou 570228, China;
dingcp1227@163.com (C.D.); zhangyunfei2020@hainanu.edu.cn (Y.Z.);
21220951320049@hainanu.edu.cn (C.C.); 21220951320094@hainanu.edu.cn (J.W.);
21210904000018@hainanu.edu.cn (M.Q.);
20213007402@hainanu.edu.cn (Y.G.); 990992@hainanu.edu.cn (L.W.)
* Correspondence: sjzhang@hainanu.edu.cn (S.Z.);
yanpluo2012@hainanu.edu.cn (Y.L.)
† These authors contributed equally to this work.

Table of contents

1. **SEM images of three HMSNs. (Fig. S1)**

2. **RT-qPCR analysis of the gene expression of an antioxidant defence-related enzyme gene PAL in cowpea roots. (Fig. S2)**

3. **Zeta potential, PDI, and average particle size of three HMSNs (Tab. S1)**

4. **Standards of disease severity grading for Fusarium wilt in cowpea plant (Tab.S2)**

5. **Primers for qRT-PCR assay (Tab. S3)**

6. **Amplification program for qRT-PCR assay (Tab. S4)**

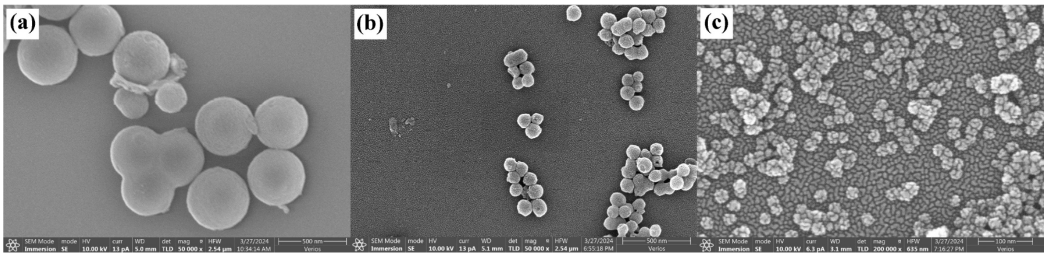

Fig. S1 HMSNs under investigation. SEM images of (a) HMSNs–406, Scale bar: 500 nm; (b) HMSNs–96, Scale bar: 500 nm; and (c) HMSNs–19, Scale bar: 100 nm.

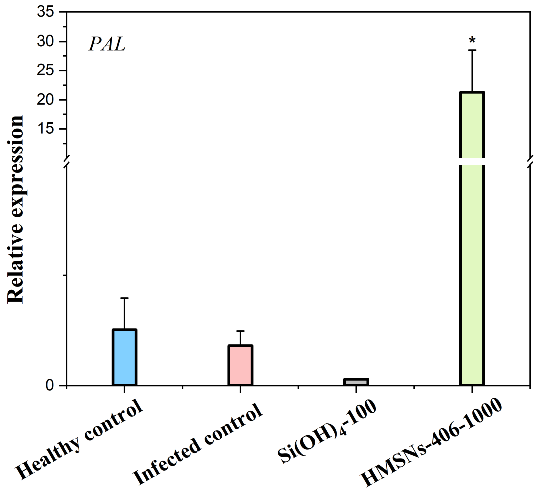

Fig. S2 RT-qPCR analysis of the gene expression of an antioxidant defence-related enzyme gene *PAL* in cowpea roots. Root samples were collected 29 days after FOP infection. *EF1b* was used as the reference gene. The healthy control represents cowpea plants growing in the noninfected soil and treated with water. Other treatments represent cowpea plants growing in the infected soil and foliarly treated with water (the infected control), $Si(OH)_4$ (100 mg/L) or HMSNs–406 (1000 mg/L). The error bars are averages and standard deviations of three replicates. Asterisks (*) represent significant differences as compared with the infected controls using the one-way ANOVA mode for significance testing with Dunnett's multiple comparisons test at $P < 0.05$.

Tab. S1 Zeta potential, PDI, and average particle size of three HMSNs

HMSNs[a]	Z-Average[b] (nm)	PDI[c]	Zeta[d] (mV)
HMSNs–19	59.26±0.19	0.14±0.01	-19.6±0.3
HMSNs–96	202.00±0.60	0.36±0.02	-33.0±1.0
HMSNs–406	690.70±12.00	0.32±0.01	-31.0±0.5

[a]HMSNs: Hollow mesoporous silica nanoparticles; [b]Z-Average: Average particle size; [c]PDI: Polydis persity index; [d]Zeta: Surface potential. Averages ± standard deviations.

Tab. S2 Standards of disease severity grading for Fusarium wilt in cowpea plant

Scale value	External leaf symptoms	Internal vascular symptoms
0	asymptomatic plants	no vascular browning
1	slight epinastic response and mild chlorosis of the lower third of the plant	up to 25% vascular browning
2	epinastic response in between 30%–50% of the leaves and moderate chlorosis in mature leaves	26–50% vascular browning
3	epinastic response in between 60%–80% of the leaves and moderate chlorosis in the middle third	51%–75% vascular browning
4	epinastic response in all the leaves of the plant, severe chlorosis and defoliation, dead plant	>75% vascular browning

Tab. S3 Primers for qRT-PCR assay

Primers	Sequence (5'-3')
PR1-F	ACTACAACTACGCTGCGAACAC
PR1-R	GTTACACCTCACTTTGGCACATC
PR5-F	GTGTTCATCACAAGCGGCAT
PR5-R	GGGAAGCACCTGGAGTCAAT
NPR1-F	TGCTCGGAAGTGTTGGATAAG
NPR1-R	GAAATCCCAGAGCGGCTAAA
EF1b-F	CCACTGCTGAAGAAGATGATGATG
EF1b-R	AAGGACAGAAGACTTGCCACTC
PAL-F	GTTTGTGAGGGAGGAGTTAGAG
PAL-R	ATGGGAGACCCTTTCCAATC
PPO-F	GCTCCTCATAACACGGTTCATA
PPO-R	GTCCTTTCTTCCTTCTCCCAAT

Tab. S4 Amplification program for qRT-PCR assay

System	Procedure
SYBR® Premix Ex TaqTM II 5.0 μL	Predegeneration at 95ºC for 10 min
PCR Forward Primer (10 um) 0.3 μL	Cycle nitiation:
PCR Reverse Primer (10 um) 0.3 μL	Degeneration at 95ºC for 15 s
cDNA solution 1.0 μL	Renaturation 60ºC for 60 s
Easy dilution (for Real Time) 3.4 μL	40 cycles
